# Autoinducer-2 May Be a New Biomarker for Monitoring Neonatal Necrotizing Enterocolitis

**DOI:** 10.3389/fcimb.2020.00140

**Published:** 2020-04-09

**Authors:** Chun-Yan Fu, Lu-Quan Li, Ting Yang, Xiang She, Qing Ai, Zheng-Li Wang

**Affiliations:** ^1^Department of Neonatal Diagnosis and Treatment Center, Children's Hospital of Chongqing Medical University, Chongqing, China; ^2^Ministry of Education Key Laboratory of Child Development and Disorders, Chongqing, China; ^3^National Clinical Research Center for Child Health and Disorders, Chongqing, China; ^4^China International Science and Technology Cooperation Base of Child Development and Critical Disorders, Chongqing, China; ^5^Children's Hospital of Chongqing Medical University, Chongqing, China; ^6^Chongqing Key Laboratory of Child Infection and Immunity, Chongqing, China

**Keywords:** autoinducer-2 (AI-2), necrotizing enterocolitis (NEC), biomarker, intestinal flora, newborn—intensive care units

## Abstract

Autoinducer-2 (AI-2) has a widely accepted role in bacterial intra- and interspecies communication. Little is known about the relationships between AI-2 and NEC. This study found that AI-2 levels in patients and in a NEC mouse model were detected using the *Vibrio harveyi* BB170 assay system. Bacterial communities of the newborns' stool microbiota (NEC acute group, NEC recovery group, control group, and antibiotics-free group) and of the NEC mouse model (NEC group and control group) were detected by high-throughput sequencing. Intestinal histopathological changes were observed after HE staining. The AI-2 level in the NEC acute group (44.75 [40.17~65.52]) was significantly lower than that in the control group, NEC recovery group and antibiotics-free group. The overall microbiota compositions of each group at the phylum level were not significantly different. The proportions of Enterococcus, Clostridium_sensu_stricto_1, Peptoclostridium, and Veillonella had significant differences among the 4 groups at the genus level. In animal experiments, the AI-2 level in feces of NEC mice (56.89 ± 11.87) was significantly lower than that in the feces of control group mice (102.70 ± 22.97). The microbiota compositions of NEC and control group mice at the phylum level were not significantly different. At the genus level, Klebsiella, Clostridium_sensu_stricto_1, and Peptoclostridium abundances in the NEC group increased significantly compared with those in the control group (*P* < 0.05). In addition, Lactobacillus, Pasteurella, and Parabacteroides abundances in the NEC group decreased significantly compared with those in the normal control group (*P* < 0.05), while Lactobacillus, Pasteurella, and Parabacteroides abundances had the opposite trend. The AI-2 concentration decreased significantly in the acute phase of NEC and increased gradually in the convalescent phase. We conclude that the concentration of AI-2 was correlated with intestinal flora disorder and different stages of disease. AI-2 may be a new biomarker for the diagnosis and monitoring of NEC.

**Trial Registry:** ClinicalTrials.gov; ChiCTR-ROC-17013746; URL: www.clinicaltrials.gov

## Introduction

Necrotizing enterocolitis (NEC) is a serious neonatal intestinal disease that most frequently affects preterm infants. NEC reportedly affects ~10% of all premature infants (Hackam et al., 2018). Neonatal care has evolved greatly over time, but despite improvements in ventilation, nutrition, and temperature regulation, NEC remains a leading cause of death from gastrointestinal disease (Warner et al., 2016). Prematurity, feeding patterns, incremental milk-feeding rates, antibiotics, intestinal ischemia, genetic factors, and intestinal bacterial colonization have been considered to be risk factors for NEC (Morrow et al., 2013). Although neonates often present with obvious clinical signs, the diagnosis of NEC can also be subtle and insidious. NEC in newborns often develops rapidly and is difficult to detect early due to a lack of specific biomarkers. Furthermore, the diagnostic modalities are always invasive or radioactive. Therefore, identifying a new biomarker for the diagnosis and monitoring of NEC that is non-invasive, non-radiative, inexpensive, and rapid is an important endeavor.

NEC does not occur in sterile animals, which proves the importance of intestinal bacterial colonization for NEC. NEC and gut bacteria are generally accepted to be causally associated (Rusconi et al., 2017). The use of antibiotics can lead to intestinal flora disorders, and many studies have reported an increased risk of NEC due to antibiotic use (Morrow et al., 2013). As Mihatsch et al. (2011) also reported, several randomized controlled trials in preterm infants have shown that probiotics can reduce the risk of NEC. Increasing evidence indicates that direct microbe-microbe interactions play a critical role in this process (Grigg and Sonnenberg, 2017), which involves density-dependent recognition of autoinducer-2 (AI-2). AI-2 is a well-known bacterial Quorum-sensing (QS) system signaling molecule that plays an important role in bacterial communication (Sun et al., 2015).

AI-2 synthase is encoded by *LuxS*, which has been shown to be able to regulate bacterial virulence, biofilm formation, and the production and release of virulence factors in numerous species (Buck et al., 2009). Many different gram-positive and gram-negative species have been reported to be able to detect the *LuxS* gene, and this phenomenon likely contributes to the interactions between bacteria inhabiting the mammalian gut (Sun et al., 2015; Thompson et al., 2015; Ismail et al., 2016). Artificially increasing the levels of AI-2 can partially counterbalance antibiotic-induced intestinal dysbiosis (Sun et al., 2015). AI-2 not only has important effects on gut colonization and probiotic functionality (Christiaen et al., 2014) but is also usually related to virulence and pathogenicity (Rees et al., 2017). Apart from these initial findings, no report on the relationship between NEC and AI-2 is available. As AI-2 is closely related to enteropathogenic bacteria and the colonization of probiotics, we speculate that AI-2 can be used as a biomarker to reflect the process of NEC.

Thus, we analyzed the bacterial composition of intestinal flora and the levels of AI-2 in infants with NEC and in control infants. The aim of the present study was to investigate whether the levels of AI-2 can help to monitor intestinal flora disorders and the intestinal inflammation of NEC.

## Materials and Methods

### Patient Selection and Sample Collection

NEC and non-NEC newborns in the Department of Neonatology at the Children's Hospital of Chongqing Medical University were enrolled in this study. NEC diagnosis was performed according to the Bell NEC standard (Hoytema van Konijnenburg et al., 2017); stage II and above cases were selected to be included in the NEC group and divided into the NEC acute group (NEC acute phase group); and NEC recovery group (NEC recovery phase). Meanwhile, non-NEC neonates that had pneumonia or hyperbilirubinemia and no clinical symptoms of the gastrointestinal tract (bloating, vomiting, bloody stool) were included in the control group. The gestational age, day age, delivery mode, feeding practices and medical conditions for each infant in the control group and antibiotic-free group (no use of antibiotics) were matched with those in the NEC group. In addition, antibiotic use in the control group was also matched with that in the NEC group.

Feces collection was performed within 24 h after the diagnosis of NEC and 3 days after initiation of feeding in the NEC acute group and in the NEC recovery group, respectively. Infants in the control group had fecal samples collected according to the matching principles mentioned before. All fecal samples were collected in sterile tubes and frozen at −80°C for subsequent measurements of AI-2 and the microbial community. Finally, 20 samples from each group were included in this study.

### Sample Preparation and AI-2 Activity Measurement

The AI-2 activity of the fecal samples was determined using the *V. harveyi* reporter strain BB170, as described previously (Raut et al., 2013; Hsiao et al., 2014). The BB170 strain (obtained from Prof. Baolin Sun, University of Science and Technology of China) was grown for 18 h at 30°C in 2216E (QDRS BIOTEC, China) medium and diluted 1:5,000 into fresh 2216E medium. Previously, frozen fecal samples were weighed to 40 mg, mixed with 1.6 ml of 2216E broth, vortexed for 5 min, and centrifuged at 4°C and 5,000 rpm for 10 min; supernatants were filtered through a 0.22 μl filter membrane (MILLIPORE, American), and the filtrate was harvested and then added to *V. harveyi* BB170 strain diluent for the AI-2 bioassay. Additionally, 20 μl of fecal filtrate, 1 μM AI-2 (Omm Scientific, American) standard solution and 2216E broth (as negative control) were added in quintuplicate to a 96-well-assay plate (Corning, American) with 180 μl of BB170 diluent to produce a final volume of 200 μl. The 96-well-assay plate was shaken at 30°C and 120 rpm. After 2.5 h, the bioluminescence intensity was measured every 30 min by a multifunction microplate reader (Thermo, USA) until the value of the negative control group was minimized. The ratio of the fluorescence of the supernatant of the fecal sample to the 1 μM AI-2 standard solution represents the relative luminescence intensity.

### Analysis of Fecal Sample Microbial Communities

Fecal microbial DNA was extracted according to the QIAamp FAST DNA Stool Mini-Kit (Qiagen, Germany) kit instructions. The V3+V4 region of the 16S rDNA gene was amplified using the universal primers 338F (5′-barcode-ACTCCTACGGGAGGCAGCA-3′) and 806R (5′-GGACTACHVGGGTWTCTAAT-3′). The polymerase chain reaction (PCR) conditions were as follows: predenaturation at 95°C for 3 min; 95°C for 30 s, 55°C for 30 s, and 72°C for 45 s for a total of 27 cycles; and, finally, 72°C for 10 min. The PCR amplification product was separated by 2% agarose gel electrophoresis, and the PCR product was recovered using an AxyPrep DNA Gel Recovery Kit (AXYGEN) and then quantitatively detected by a QuantiFluor^TM^-ST Blue Fluorescence System (Promega). The library was built and sequenced according to the Illumina MiSeq platform-related process. The obtained raw data were optimized to remove bases with a tail mass value of 20 or less, to filter reads with an overlap length >10 bp, to filter reads at a mismatch ratio >0.2 in the overlap region of the splicing sequence, and to remove reads with a mismatched barcode sequence. The optimized sequence was divided into operational taxonomic units (OTUs) by Usearch (version 7.0), and statistical analysis of the biological information was usually performed at the OTU of 97% of similarity.

### Animal Experiments

#### Neonatal Necrotizing Enterocolitis Mouse Model

Newborn C57BL/6J mice were purchased from the Animal Experiment Center of Chongqing Medical University (Chongqing, China). The NEC mouse model was previously described by Garg et al. (2015); Li et al. (2017), and Xiao et al. (2018) and was adopted here with some modifications: 7-day-old C57BL/6 newborn mice were randomly separated into two groups. The control group was breast-fed with their mothers, and the experimental group was fed with formula milk (2 g of Similac Advance in 10 ml of 33% Esbilac puppy milk replacer) (Zeng et al., 2017) at 30 μl/1 g body weight every 4 h with a silicone tube (1.9 Fr) for 3 days. These pups were also stressed three times a day with hypoxia treatments (100% nitrogen gas for 1 min) and cold stimulation (4°C) for 10 min; the control group did not undergo these interventions.

#### Histology Examination

Both the control and NEC group animals were decapitated 3 days after NEC induction. After the intestine was removed from the body, a 1-cm section of the distal ileum was cut and fixed in a 4% paraformaldehyde solution. Next, the tissue sample was dehydrated, embedded in paraffin, and cut into 4 μm slices. Subsequently, 4 μm tissue slices were stained with hematoxylin and eosin (HE). Finally, we observed histopathological changes under an optical microscope. The scoring system was graded as follows: normal (0); epithelial cell lifting or separation (1); necrosis to the midvillous level (2); necrosis of the entire villus (3); and transmural necrosis (4). The intestinal tissue injury was graded by a blinded evaluator, and a score of ≥2 points was considered positive for NEC (Dvorak et al., 2008; Yu et al., 2009).

#### Mouse Intestinal Content Collection and AI-2 Activity Measurement

The ileum and colon of the mice were lavaged with 400 μl of 2216E broth, and the intestinal contents were collected in sterile tubes before vortex oscillating, centrifuging, and filtering as previously described. Then, the supernatant filtrate was collected for an AI-2 bioassay, and the sediment was stored at −80°C. The AI-2 standard solution was diluted to 1 μM, 100 nM, and 10 nM. Finally, the AI-2 activity assay was performed as described above.

#### Analysis of Intestinal Microbial Community

The sediment from the previous step was used for intestinal microbial community analysis, as described above.

### Statistical Analysis

SPSS version 24.0 (SPSS Inc., USA) was used to perform statistical analyses. The measurement data were tested for normal distribution. The data showing a normal distribution were expressed as the mean ± standard deviation (SD) and analyzed by a paired *t*-test or independent-sample *t*-test. Skewed data were analyzed by the Mann-Whitney *U*-test or Kruskal-Wallis test and described by the median and interquartile range (IQR).

## Results

### Clinical Characteristics

**Figure 1:**
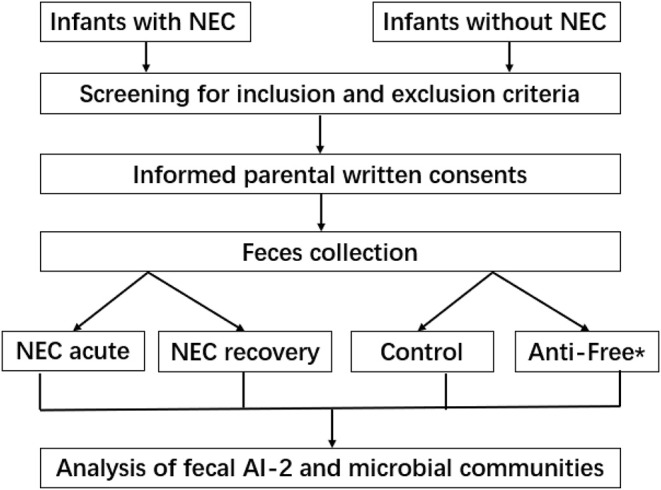
Flow diagram of the study protocol. *Anti-free, the antibiotic-free group.

Clinical background information of the enrolled infants is shown in Table 1. The subjects in the three groups—the NEC group, antibiotic-free group and control group—were sufficiently case-matched, and the flowchart is shown in Figure 1. No significant differences in the mode of delivery, type of feeding, gestational age, and gender ratio were noted between the NEC group and the control group.

**Table 1:** Patient characteristics.

**Variables**	**Anti-free (*n* = 20)**	**Control (*n* = 20)**	**NEC (*n* = 20)**	^*^P-value
Age, (*x* ± s) day	11.80 ± 6.60	12.35 ± 6.59	12.45 ± 6.72	0.265
GA, (*x* ± s) week	37.85 ± 2.35	37.65 ± 2.25	37.60 ± 2.62	0.17
Male, % (*n*)	55% (11)	55% (11)	11/9	1
Breast feeding, % (*n*)	20% (4)	15% (3)	10% (2)	0.38/0.63
Vaginal delivery, % (*n*)	40% (8)	40% (8)	40% (8)	1
Procalcitonin M (P_25_, P_75_) ng/ml	0.08 (0.06, 0.10)	0.34 (0.10, 3.34)	1.47 (0.09, 15.68)	0.00
CRP > 8 mg/L, % (*n*)^*^	0% (0)	45% (9)	50% (10)	0.00
Bloody stool, % (*n*)	0% (0)	0% (0)	80% (16)	0.00
Vomiting	0% (0)	0% (0)	30% (6)	0.00
Intrahepatic or portal venous gas, % (*n*)	0% (0)	0% (0)	100% (20)	0.00

* *CRP, C-reactive protein*.

### AI-2 Assays of Neonatal Feces

**Figure 2:**
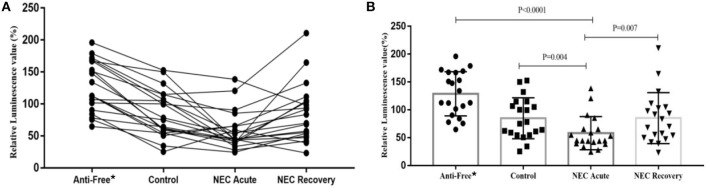
The relative biofluorescence values of AI-2 in feces from each group. **(A)** Bar graph of the relative biofluorescence values of AI-2 in feces and **(B)** Line graph of the relative biofluorescence values of AI-2 in feces. *Anti-free, antibiotic-free group.

To determine whether the level of AI-2 varies with disease progression, the relative levels of AI-2 in the 4 groups were analyzed. As shown in Figure 2, the level of AI-2 in the NEC acute group (44.75 [40.17~65.52]) was significantly lower than that in the control group (76.65 [55.85~112.77], *P* = 0.004) and antibiotic-free group (123.33 [93.15~167.21], *P* < 0.0001).

Furthermore, the level of AI-2 in the antibiotic-free group was significantly higher than that in the control group (*P* < 0.001). The level of AI-2 in the NEC recovery group (76.68 [50.99~104.48]) was significantly higher than that in the NEC acute group (44.75 [40.17~65.52], *P* = 0.007), but 3 NEC infants showed the opposite trend (lower or almost the same compared with their recovery phase). One of the 3 infants suffered NEC recurrence, and another one developed milking slowness and abdominal distension.

### Diversity Analysis of the Microbiota

Fecal samples collected from the infants were amplified by the universal bacterial 16S rRNA primers. The positive PCR products were sequenced by the Illumina MiSeq high-throughput platform, and 7,15,040 effective sequences (through quality control) were generated, with an average of 8,938 sequences per sample.

The Shannon index values of the NEC acute and NEC recovery groups tended to be lower than those of the control group and antibiotic-free group. Nevertheless, no significant difference was found among the 4 groups (Table 2).

**Table 2:** The Shannon index of each group at the phylum and genus levels, *M* (*P*_25_~*P*_75_).

**Shannon index**	Anti-free^*^	**Control**	**NEC acute**	**NEC recovery**	***H*-value**	***P***
Phylum level	0.67 (0.36~0.84)	0.47 (0.35~0.72)	0.51 (0.20~0.62)	0.32 (0.08~0.62)	3.94	0.27
Genus level	0.85 (0.61~1.20)	0.93 (0.64~1.09)	0.83 (0.71~1.09)	0.68 (0.23~1.04)	3.90	0.27

* *Anti-free, antibiotic-free group*.

### Composition Analysis of the Microbiota

**Figure 3:**
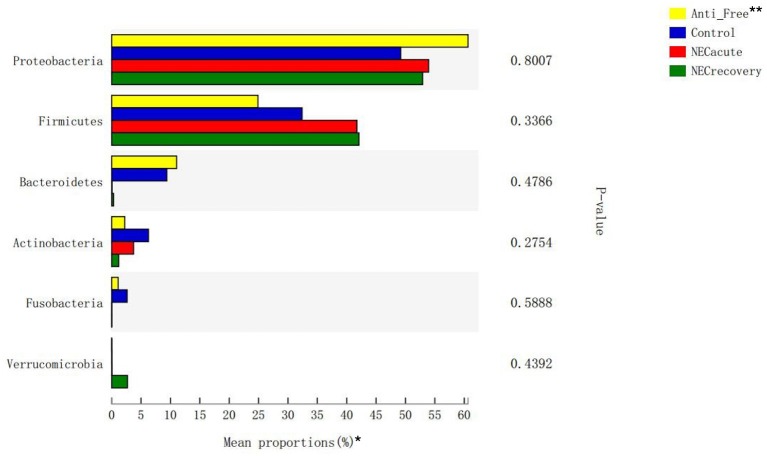
Differences in flora composition in different groups at the phylum level. *The top six phyla are shown in the figure. **Anti-free, antibiotic-free group.

**Figure 4:**
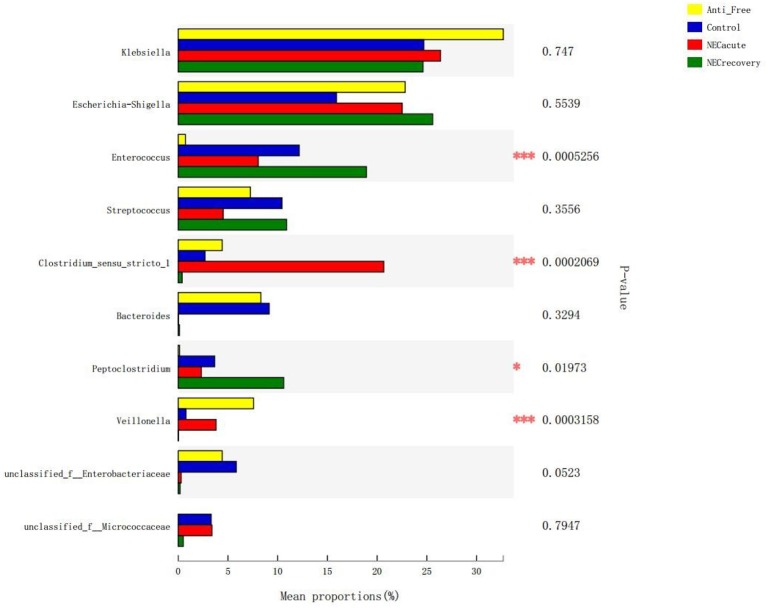
Differences in flora composition in different groups at the genus level. Anti-free, antibiotic-free group (*P < 0.05; ***P < 0.001).

The overall microbiota compositions of each group at the phylum and genus levels are shown in Figures 3, 4. At the phylum level, the prevalence of *Firmicutes* was higher in the acute and recovery phases of NEC than that in the control group and antibiotic-free group, while *Bacteroides* tended to decrease in the acute and recovery phases of NEC, but no significant difference was observed (*P* > 0.05). As shown in Figure 3, *Enterococcus* increased in the acute and recovery NEC stage groups (*P* = 0.0005) compared with in the antibiotics-free group. *Clostridium_sensu_stricto_1* increased significantly in the acute stage of NEC (*P* = 0.0002); *Peptoclostridium* had the highest average proportion in the NEC recovery group and the lowest average proportion in the antibiotics-free group (*P* = 0.02); and *Veillonella* had the highest average proportion in the antibiotics-free group and the lowest average proportion in the NEC recovery group (*P* = 0.003).

### General Situation of Model Mice

**Figure 5:**
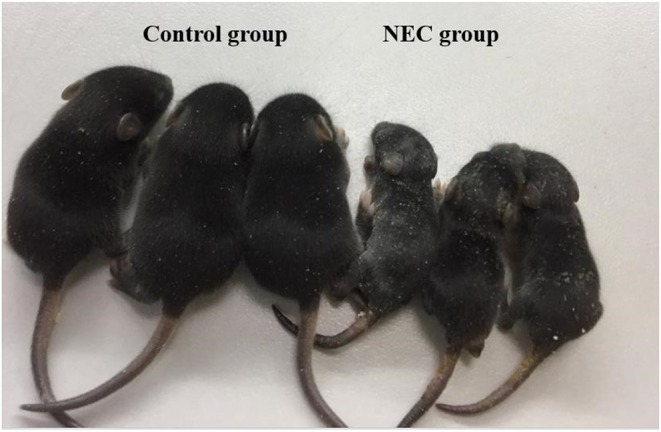
The physical growth of mice in the NEC group and mice in the control group.

**Figure 6:**
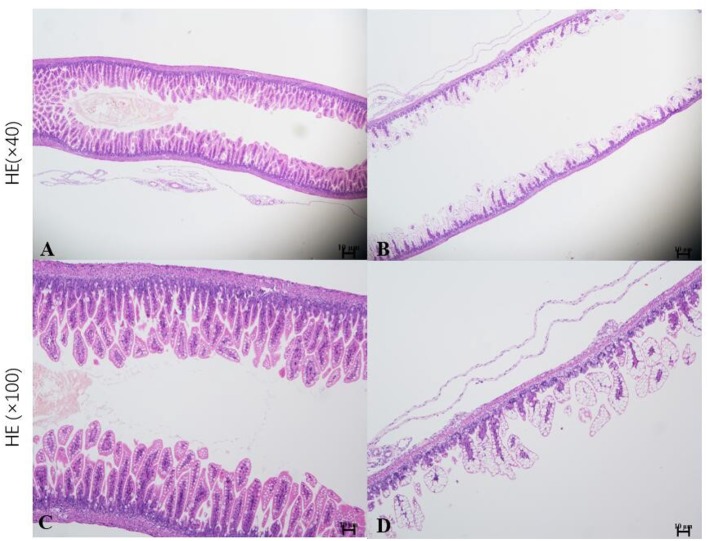
Histopathological analysis of the intestinal tissue in mice. *Sections of intestinal tissue stained with hematoxylin and eosin are shown. **(A,C)** Control group and **(B,D)** NEC group.

**Figure 7:**
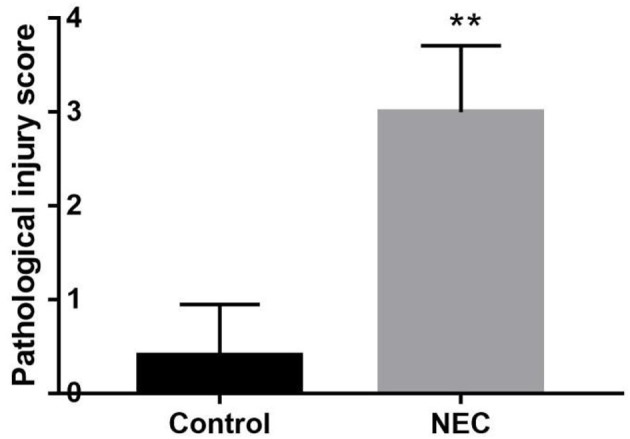
Comparison of intestinal histopathological injury scores between NEC group mice and control group mice **P < 0.05.

Neonatal mice in the control group grew well, reacted sensitively, moved normally, fed and defecated normally, and had plentiful subcutaneous fat. Neonatal mice in the NEC group gradually developed gastric retention, feeding difficulties, abdominal distention, diarrhea and even black stool, and hematochezia from the second day of modeling, accompanied by an obvious decrease in activity, poor reaction, and small size (Figure 5). As shown in Figure 6, the intestinal barrier was destroyed, and intestinal wall tissue necrosis occurred in the NEC group, while the intestinal wall barrier in the control group was intact without tissue necrosis. The pathological injury score results showed that the pathological injury score of intestinal tissue in the NEC group was significantly higher than that in the control group (Figure 7).

### AI-2 Assays of Mouse Feces

**Figure 8:**
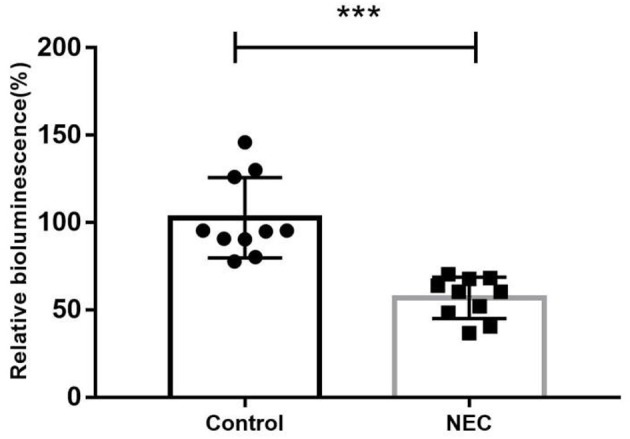
The relative biofluorescence values of AI-2 in feces from two groups of newborn mice (***P < 0.0001).

As shown in Figure 8, the AI-2 level in the feces of NEC mice (56.89 ± 11.87) was significantly lower than that in the control group (102.70 ± 22.97), which was consistent with the clinical findings above.

### Composition Analysis of the Microbiota in the NEC Mouse Model

**Figure 9:**
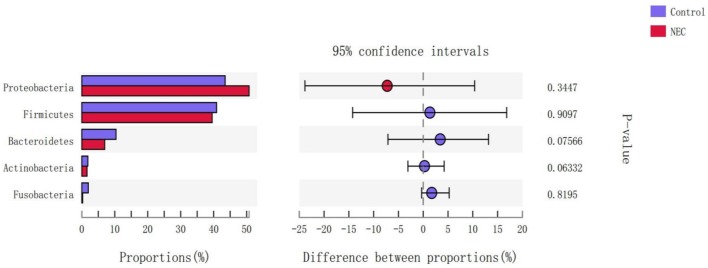
Differences in the flora composition of mice at the phylum level.

**Figure 10:**
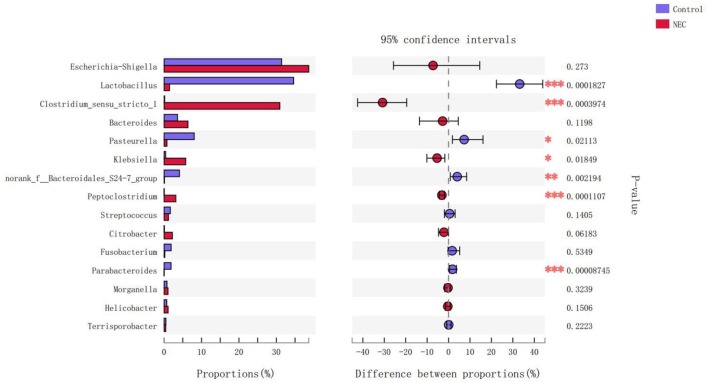
Differences in the flora composition of mice at the genus level (*P < 0.05; **P < 0.01; ***P < 0.001).

At the phylum level (Figure 9), *Proteus* in the NEC mouse group was more abundant than that in the control group, while *Bacteroides* in the NEC group was less abundant than that in the normal control group, but no significant difference was noted (*P* > 0.05). At the genus level (Figure 10), *Klebsiella, Clostridium_sensu_stricto_1*, and *Peptoclostridium* abundance levels in the NEC mouse group increased significantly compared with those in the control group (*P* < 0.05). In addition, *Lactobacillus, Pasteurella*, and *Parabacteroides* abundances in the NEC group decreased significantly compared with those in the control group (*P* < 0.05).

## Discussion

NEC is a serious intestinal disorder of newborns, with high mortality and morbidity. In clinical practice, the main examinations for monitoring diseases include white blood cell count, procalcitonin levels, C-reactive protein levels, abdominal imaging and so on. Our study further revealed that AI-2 was significantly associated with the occurrence and recovery of NEC and that the level of AI-2 in NEC patients in the acute stage was lower than that in the controls and NEC patients in the recovery stage.

Although no single pathogen or pathogenic microbial community has been consistently identified to be associated with NEC, abnormal intestinal colonization has long been thought to contribute to NEC in newborns (Anatoly et al., 2013). Compared with previous studies that relied on culture or gel-based technology for microbial identification, the application of second-generation sequencing technology has improved our ability to evaluate this hypothesis (Morrow et al., 2013; Rusconi et al., 2017). When gut microbial diversity is examined in NEC, irrespective of the community composition, the results are mixed between studies. Many studies have reported a decrease in stool microbial diversity between NEC and control infants (McMurtry et al., 2015; Stewart et al., 2016; Ward et al., 2016; Warner et al., 2016), whereas some studies have found no difference (Mai et al., 2011; Morrow et al., 2013; Torrazza et al., 2013). As shown in Table 2, no difference was identified in the abundance of Proteobacteria at the phylum level (Figure 3) or in the Shannon index of each group at the phylum and genus levels. Furthermore, there was a significant difference in the abundance of *Enterococcus, Clostridium_sensu_stricto_1, Veillonella*, and *Peptoclostridium* at the genus level (Figure 4). The NEC recovery group had the lowest proportion of *Veillonella* and the highest proportion of *Peptoclostridium*. Gupta et al. (1994) also reported that *Enterococcus* spp*., Staphylococcus epidermidis*, and *Escherichia coli* were the most common aerobic bacterial species isolated, but no single pathogen was associated with the occurrence of NEC. A recent study reported that the proportion of *Clostridium_sensu_stricto_1* was increased in the pediatric diarrheic intestine (Wang et al., 2018). Wang Y. et al. (2017) also reported that, a high-grain diet dynamically increased the relative abundance of *Clostridium_sensu_stricto_1* and induced mucosal injuries in the colon of sheep. Quinoa consumption has been reported (Liu et al., 2018) to significantly alleviate DSS-induced dysbiosis as indicated by decreased abnormal expansion of the phylum Proteobacteria and decreased overgrowth of the genus *Peptoclostridium*. We also found that the abundances of *Enterococcus, Peptoclostridium*, and *Veillonella* were significantly different between the antibiotics-free group and the control group, which indicated that antibiotics have a great influence on the intestinal flora of newborns.

AI-2 is a bacterial interspecies signaling molecule that plays an important role in the regulation of virulence factor production in pathogenic gram-negative and gram-positive bacteria (Pereira et al., 2013; Christiaen et al., 2014). AI-2 can be detected by bioluminescence method, only 50-100 mg feces are needed, a little bacterial culture medium and conventional bacterial culture equipment are used, and the detection can be completed in 3–4 h. It affects bacterial toxin production, biofilm formation, intestinal dysbiosis, motility, adherence to epithelial cells, and metabolism (Pereira et al., 2013; Sun et al., 2015; Thompson et al., 2015; Ismail et al., 2016; Wang et al., 2016). In the present study, there was a reduction in AI-2 levels in the NEC acute group, which were increased in the NEC recovery group. Furthermore, the AI-2 level of the control group was relatively lower than that of the antibiotics-free group, which indicated that antibiotic treatment had an effect on AI-2 levels.

Interestingly, the AI-2 levels of three NEC acute subjects were lower than those of the recovery phase subjects, one of whom had a recurrence of NEC and another of which had mild abdominal distension, poor digestion of milk, and a slow feeding rate. As Sun et al. (2015) also reported, antibiotic treatment leads to disruption of the normal composition of the microbiota and reductions in AI-2 levels and AI-2-producing bacteria, resulting in enhanced virulence gene expression by pathogens, while artificially increasing the levels of AI-2 partially reverses dysbiosis and reduces virulence gene expression (Sun et al., 2015). As Hsiao et al. (2014) also reported, *R. obeum* AI-2 reduces *Vibrio* colonization/pathogenicity and autoinducers and/or other mechanisms to limit colonization with *V. cholerae* or conceivably other enteropathogens. We speculate that when NEC occurs, the concentration of AI-2 decreases, the inhibitory effect of AI-2 on the virulence of pathogenic bacteria is reduced, and probiotic colonization is blocked. When NEC recovers, the AI-2 concentration increases, which may inhibit the virulence of pathogenic bacteria and promote the colonization of probiotics. These findings may indicate that AI-2 is related to intestinal flora disorders, which can also reflect bacterial pathogenicity and disease recovery in NEC to some extent.

Usually, we monitor NEC progress through WBC (white blood cell) counts, PCT (procalcitonin) levels, C-reactive protein levels, abdominal imaging, and so on. Recently, there have been many publications examining NEC biomarkers in the past decades, such as cytokines (IL-8, TGF-β, IL-1RA, IL-1β), calprotectin, the non-protein amino acid citrulline, urine intestinal fatty acid-binding protein (IFABP), serum amyloid A (SAA), fecal calprotectin, the EpCAM/MMp7 ratio, a serum protein panel, volatile organic compounds, acylcarnitines, fibrinogen-g dimers, and so on (Rusconi et al., 2017; Hackam et al., 2018). However, no biomarkers have achieved widespread clinical application, and existing biomarkers cannot reflect intestinal flora disorder (Anatoly et al., 2013; Neu, 2014; Garg et al., 2018); furthermore, such examinations are usually invasive or radioactive (Stoll et al., 2015; Rusconi et al., 2017).

Methods such as direct-from-stool amplification and sequencing of the 16S ribosomal RNA subunit DNA or whole-genome shotgun (WGS) sequencing help us to identify microbial community members and distributions. However, these methods are very expensive and time-consuming (Rusconi et al., 2017). AI-2 has been proposed to promote interspecies bacterial communication in the mammalian gut (Ismail et al., 2016) and not only plays a crucial role in gut colonization and probiotic functionality (Christiaen et al., 2014) but is also related to intestinal dysbiosis (Bivar, 2018; Zhao et al., 2018). As in shown in Figures 2, 3, we found that the level of AI-2 is affected by antibiotics, and that AI-2 can change before the proportion of bacteria changes significantly. In addition, the AI-2 examination was non-invasive, fast, inexpensive, and radiation free and may therefore play an important role in the diagnosis and monitoring of diseases.

Based on the current findings, the NEC mouse model showed similar changes in intestinal flora disorder and AI-2 concentrations, as shown in Figures 6–8. The changes in intestinal flora, AI-2 level, and pathology in the NEC mouse model were similar to those in infants with NEC. Interestingly, *Clostridium_sensu_stricto_1*, which was also much more abundant in NEC infants of the acute stage than in the other infants (Figure 10), was significantly more abundant in the NEC mouse group than in the control mouse group. *Clostridium* is one of the most common anaerobes identified from the common fecal microflora of preterm infants (Laurent et al., 2012), and *Clostridium_sensu_stricto_1* in particular was considered to be associated with NEC (Schonherr-Hellec and Aires, 2019). In studies of the intestinal flora of animals, *Clostridium_sensu_stricto_1* has been reported to have a detrimental effect on the colonic health of sheep (Wang J. et al., 2017; Wang Y. et al., 2017).

Our study suggested that AI-2 has obvious changes associated with the onset and progression of NEC in both animal experiments and clinical studies, so AI-2 may be a new marker for monitoring NEC.

In summary, the present results reveal that the AI-2 concentration decreased significantly in the acute phase of NEC and increased gradually in the convalescent phase of NEC. The concentration of AI-2 was correlated with intestinal flora disorder and different stages of disease and changed before the proportion of bacteria changed significantly. AI-2 may be a new biomarker for monitoring NEC.

## Data Availability Statement

The datasets generated for this study are available on request to the corresponding author.

## Ethics Statement

The studies involving human participants were reviewed and approved by the Ethics Committee of the Children's Hospital of Chongqing Medical University. Written informed consent to participate in this study was provided by the participants' legal guardian/next of kin. The animal study was reviewed and approved by Institutional Animal Care and Use Committee (IACUC) at the University of Chongqing Medical University.

## Author Contributions

L-QL and Z-LW contributed to conceptualization, funding acquisition, and supervision. C-YF and TY helped with data curation. L-QL, C-YF, TY, and XS worked on the formal analysis. C-YF, L-QL, TY, XS, and Z-LW carried out the investigation. C-YF, XS, Z-LW, and QA contributed to the methodology. C-YF and Z-LW were responsible for project administration and writing the original draft. TY and QA contributed to resources. TY was responsible for the validation. Z-LW and L-QL reviewed and edited the manuscript.

### Conflict of Interest

The authors declare that the research was conducted in the absence of any commercial or financial relationships that could be construed as a potential conflict of interest.
